# The molecular characteristics of spinal cord gliomas with or without H3 K27M mutation

**DOI:** 10.1186/s40478-020-00913-w

**Published:** 2020-03-30

**Authors:** Rui-Chao Chai, Yao-Wu Zhang, Yu-Qing Liu, Yu-Zhou Chang, Bo Pang, Tao Jiang, Wen-Qing Jia, Yong-Zhi Wang

**Affiliations:** 1 Department of Molecular Neuropathology, Beijing Neurosurgical Institute, Capital Medical University, No. 119 South 4th Ring West Road, Fengtai, District, Beijing, 100070 People’s Republic of China; 2 China National Clinical Research Center for Neurological Diseases, Beijing, China; 3 Chinese Glioma Genome Atlas Network (CGGA), Beijing, China; 4 Department of Neurosurgery, Beijing Tiantan Hospital, Capital Medical University, No. 119 South 4th Ring West Road, Fengtai District, Beijing, 100070 People’s Republic of China

**Keywords:** Spinal cord, Glioma, H3 K27M, *TERT* promoter, *BRAF* V600E

## Abstract

Due to the rare incidence of spinal cord astrocytomas, their molecular features remain unclear. Here, we characterized the landscapes of mutations in H3 K27M, isocitrate dehydrogenase 1 (*IDH1*) R132H, *BRAF* V600E, and the *TERT* promoter in 83 diffuse spinal cord astrocytic tumors. Among these samples, thirty-five patients had the H3 K27M mutation; this mutant could be observed in histological grade II (40%), III (40%), and IV (20%) astrocytomas. *IDH1* mutations were absent in 58 of 58 cases tested. The *BRAF* V600E mutation (7/57) was only observed in H3-wildtype astrocytomas, and was associated with a better prognosis in all histological grade II/III astrocytomas. *TERT* promoter mutations were observed in both H3 K27M-mutant (4/25) and -wildtype (9/33) astrocytomas, and were associated with a poor prognosis in H3-wildtype histological grade II/III astrocytomas. In the 2016 WHO classification of CNS tumors, H3 K27M-mutant diffuse midline gliomas, including spinal cord astrocytomas, are categorized as WHO grade IV. Here, we noticed that the median overall survival of histological grade II/III H3 K27M-mutant cases (*n* = 28) was significantly longer than that of either the total histological grade IV cases (*n* = 12) or the H3 K27M-mutant histological grade IV cases (*n* = 7). We also directly compared H3 K27M-mutant astrocytomas to H3-wildtype astrocytomas of the same histological grade. In histological grade II astrocytomas, compared to H3-wildtype cases (*n* = 37), H3 K27M-mutant patients (*n* = 14) had showed a significantly higher Ki-67-positive rate and poorer survival rate. However, no significant differences in these parameters were observed in histological grade III and IV astrocytoma patients. In conclusion, these findings indicate that spinal cord astrocytomas are considerably different from hemispheric and brainstem astrocytomas in terms of their molecular profiles, and that the histological grade cannot be ignored when assessing the prognosis of H3 K27M-mutant spinal cord astrocytomas.

## Introduction

Currently, accurate molecular pathological information is vital for the precise diagnosis and clinical management of gliomas in the brain; their key molecular features, including isocitrate dehydrogenase (IDH) mutations, 1p/19q co-deletion, and the H3 K27M mutation, are included in the World Health Organization (WHO) 2016 classification of tumors of the central nervous system [19, 24, 25]. Although novel molecular markers are constantly being discovered for brain gliomas, the molecular characteristics of gliomas and their prognostic value for spinal cord gliomas are still largely unknown, except for the H3 K27M mutation [5, 9, 17, 27, 29, 34]. Currently, our understating of spinal cord astrocytomas is largely based on advances in their intracranial counterparts, as the low incidence of spinal cord astrocytomas leads to difficulties in collecting sufficient samples to run adequate analyses. However, genetic alterations and the molecular biological profile of spinal cord gliomas seem to be distinct from those of their brain counterparts [1, 2, 13, 27, 33, 34, 37, 38].

According to the 2016 WHO classification, diffuse midline gliomas with an H3 K27M mutation in either H3F3A or HISTIH3B/C are considered a novel entity known as “diffuse midline glioma, H3 K27M-mutant” [24]. This novel entity is categorized as a grade IV glioma regardless of its other histological features, and includes tumors in different locations, such as the thalamus, pons, and spinal cord [21, 24, 33]. In general, H3 K27M mutation is associated with a poor outcome [22]. However, this conclusion is mainly based on studies of diffuse intrinsic pontine gliomas (DIPG) or pan-midline gliomas, while 80% of DIPG cases are H3 K27M-mutant gliomas [12, 22, 33].

Whether the prognostic value of the H3 K27M mutation is influenced by tumor location remains uncertain. The Consortium to Inform Molecular and Practical Approaches to CNS Tumor Taxonomy—Not Official WHO—have clarified the diagnostic criteria for “Diffuse midline glioma, H3 K27M-mutant,” and suggested that this term should be reserved for tumors that are diffuse (i.e., infiltrating), midline (e.g., thalamus, brainstem, spinal cord, etc.) gliomas with the H3 K27M mutation, and should not be applied to other tumors with the H3 K27M mutation [23]. A previous study of 77 midline gliomas indicated that H3 K27M-mutant gliomas have a universally fatal prognosis, independent of tumor location [21]. In contrast, a separate study of 120 patients indicated that the H3 K27M mutation is only associated with a poor prognosis in infratentorial gliomas, but not in supratentorial gliomas [33]. Although the H3 K27M mutation appeared to be associated with poor survival rates for spinal cord gliomas (less than 30 cases, in which the H3 K27M mutation was identified in 50–60% of tumors) in these studies, another study on twenty-five 2016 WHO grade IV spinal cord gliomas (including 20 with the H3 K27M mutation) indicated that the H3 K27M mutation was associated with a better prognosis [38]. Thus, the prognostic value of the H3 K27M mutation for spinal cord glioma remains controversial. Because many of the previous studies included less than 30 spinal cord glioma cases, it is vital to clarify their molecular features and prognostic value, especially for the H3 K27M mutation, in a large-scale study of spinal cord astrocytomas.

Here, we retrospectively analyzed the clinical and basic molecular pathological features of 83 patients with spinal cord astrocytomas diagnosed between 2011 and 2018 in the Beijing Tiantan Hospital. In addition to clinical characteristics such as age, gender, anatomic tumor location, and extent of tumor resection, the H3 K27M-mutant status of all patients was identified. We also analyzed the distributions of *IDH1* R132H mutation, *BRAF* V600E mutation, *TERT* promoter mutation, and their prognostic value in 58 cases (57 for *BRAF* V600E mutation). Importantly, we also directly compared the clinicopathological features and prognoses of H3 K27M-mutant and H3-wildtype astrocytomas in each of the grade II, III, and IV samples.

## Materials and methods

### Patients

We retrospectively collected the data of 83 patients with diffuse astrocytic tumors in the spinal cord between 2011 and 2018 in the Department of Neurosurgery at Beijing Tiantan Hospital, China (Table 1). In the present study, the following inclusion criteria were used: (A) all patients included in the current study had a defined histopathological diagnosis of glioma according to the 2016 WHO classification; (B) either the H3 K27M-mutant status or formalin-fixed/paraffin-embedded (FFPE) samples were available; and (C) follow-up information for the patients was available.

**Table 1:** Characteristics of patients with spinal cord astrocytoma in this study

		Total	Total (Grade II–IV)		*P*-value
			H3 wildtype	H3 K27M-mutant	
Number		83	48	35	
Age (year)	Median	31(6–63)	30(6–63)	35(9–52)	n.s
Gender					n.s
	male	49(59%)	31(65%)	18(51%)	
	female	34(41%)	17(35%)	17(49%)	
Histological grade					**0.0068**
	II	51(61%)	37(77%)	14(40%)	
	III	20(24%)	6(13%)	14(40%)	
	IV	12(14%)	5(10%)	7(20%)	
Location					n.s
	C	29(35%)	19(40%)	10(29%)	
	C-T	13(16%)	6(13%)	7(20%)	
	T	34(41%)	19(40%)	15(43%)	
	T-L	7(8%)	4(8%)	3(9%)	
Resection					**< 0.0001**
	GTR	50(60%)	38(79%)	12(34%)	
	STR	16(19%)	6(13%)	10(29%)	
	OB	17(20%)	4(8%)	13(37%)	
Radio					n.s
	yes	53(64%)	30(63%)	23(66%)	
	no	18(22%)	12(25%)	6(17%)	
	unknown	12(14%)	6(13%)	6(17%)	
Chemo					**0.0484**
	yes	23(28%)	10(21%)	13(37%)	
	no	47(57%)	32(67%)	15(43%)	
	unknown	13(16%)	6(13%)	7(20%)	
IDH					n.s
	wildtype	58(70%)	33(69%)	25(71%)	
	mutant	0(0%)	0(0%)	0(0%)	
	unknown	25(30%)	15(31%)	10(29%)	
TERT promoter					n.s
	wildtype	45(54%)	24(50%)	21(60%)	
	mutant	13(16%)	9(19%)	4(11%)	
	unknown	25(30%)	15(31%)	10(29%)	
BRAF V600E					**0.0141**
	wildtype	50(60%)	26(54%)	24(69%)	
	mutant	7(8%)	7(15%)	0(0%)	
	unknown	26(31%)	15(31%)	11(31%)	
Ki-67					**< 0.0001**
	< 10%	44(53%)	37(63%)	7(20%)	
	≥10%	46(55%)	18(33%)	28(80%)	
	unknown	3(4%)	3(4%)	0(0%)	
Survival (Median OS, months)		40.13	Und	20.7	**< 0.0001**

*C* cervical vertebrae, *C-T* cervicothoracic vertebrae, *T* thoracic vertebrae, *T-L* thoracolumbar vertebrae, *GTR* gross total resection, *STR* subtotal resection, *OB* open biopsy, *Radio* Radiotherapy, *Chemo* Chemotherapy

Significant *P*-values are indicated in bold text

### Clinical information collection

The basic clinical information of patients was collected and summarized (Table 1). The extent of resection was estimated by evaluating post-surgery magnetic resonance images, and the extent of resection was classified as gross total resection (GTR, ≥ 90%), subtotal resection (STR, ≥ 50% and < 90%), or open biopsy (OB, < 50%). Patients who received radiotherapy were treated with postoperative conventional adjuvant radiation therapy at a total dose of 40–50 Gy. The adopted chemotherapy regimen was temozolomide (TMZ) at a daily dose of 75 mg/m^2^ during radiotherapy, or 5 consecutive days per treatment cycle with 4 weeks per cycle at a dose of 150–200 mg/m^2^.

### Evaluation of histological and molecular pathological features

**Fig. 1:**
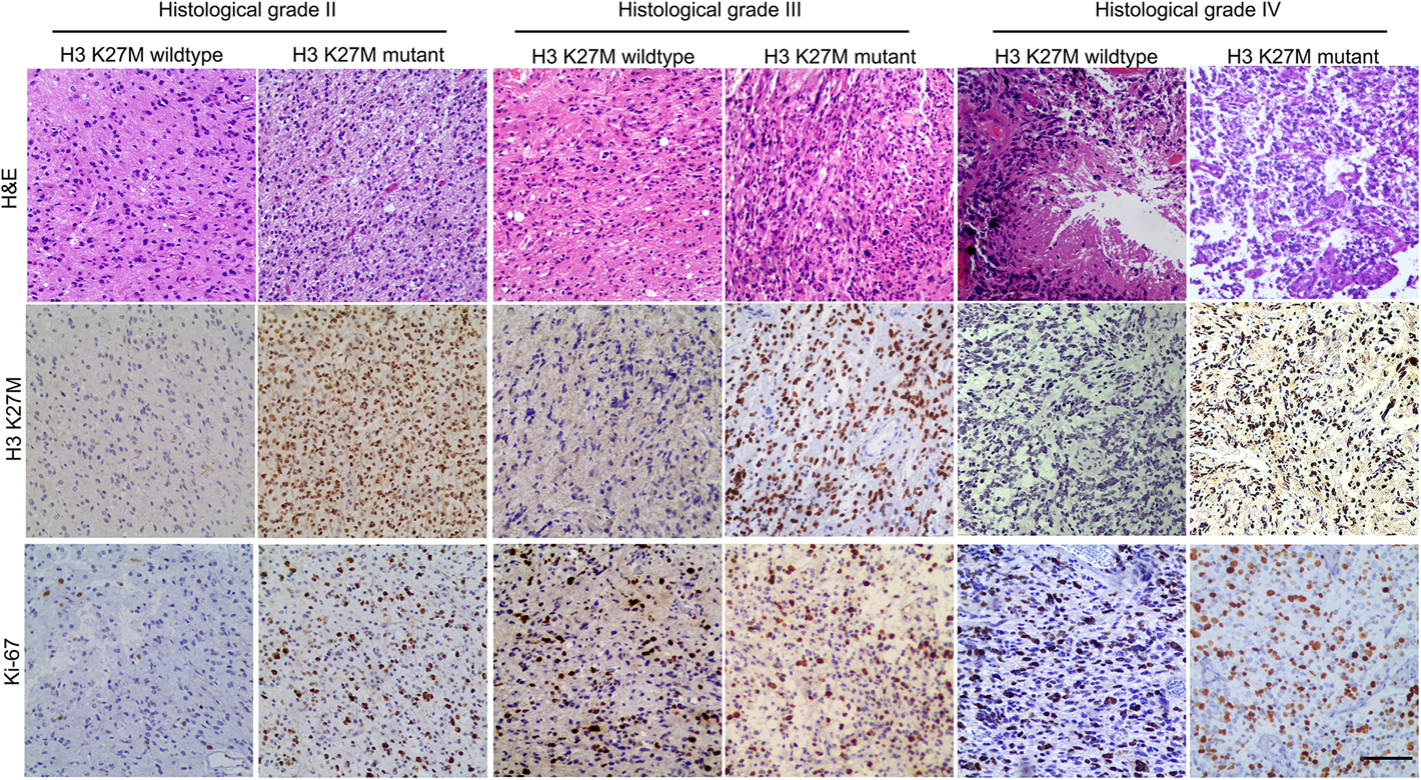
Histological features, the H3 K27M-mutant status, and Ki-67 staining images of spinal cord astrocytomas. Representative images of spinal cord glioma samples with different histological grades and the H3 K27M-mutant status. Histological grade was determined based on hematoxylin and eosin (H&E) staining. The presence of the H3 K27M mutation and Ki-67 expression were identified by immunohistochemical staining. Scale bar = 50 μm

As shown in Fig. 1, the histopathological grade was determined by the routine evaluation of FFPE samples with hematoxylin and eosin (H&E) staining based on the 2016 WHO classification [24]. All slices were reviewed by at least two experienced neuropathologists. Fifty-one cases with the features that tumor cells with cytological atypia alone were considered histological grade II; 20 cases that also showed features of anaplasia and mitotic activity were considered histological grade III; and 12 samples that additionally showed microvascular proliferation and/or necrosis (including focal microvascular proliferation and/or necrosis) were classified as histological grade IV. Immunohistochemistry with the corresponding antibodies was used to evaluate the H3 K27M-mutant status (ABE419; Millipore, Billerica, MA, USA; 1:800) and Ki-67 expression status (MIB-1; Labvision, Fremont, CA, USA; 1:50), and the proportion of Ki-67-positive nuclei was calculated manually by neuropathologists. Based on the integrated diagnosis of the 2016 WHO classification, 28 H3 K27M-mutant histological grade II/III astrocytomas were also considered 2016 WHO grade IV.

The *IDH1* R132H, *TERT* promoter C228T and C250T, and *BRAF* V600E mutations were determined by pyrosequencing (PSQ) after PCR amplification in 66 cases where sufficient DNA could be extracted from FFPE samples (QIAmp DNA Mini Kit; Qiagen, Hilden, Germany); the PCR and sequencing primers used are listed in Supplementary Table 1. *MGMT* promoter methylation was also assessed by PSQ with the PyroMark Q24 MGMT kit on a PyroMarker Q24 instrument (Qiagen), as previously described [7]. Due to the limited DNA materials, we did not test the status of the non-canonical *IDH* mutations, such as non-*IDH1* R132H and *IDH2* mutations.

### Statistical analysis

Statistical analysis was performed using SPSS (IBM, Armonk, NY, USA) and GraphPad Prism 7 (GraphPad Software, La Jolla, CA, USA).

Gliomas were classified into two subgroups based on their H3 K27M-mutant status in all gliomas or according to the specific histological grade. A nonparametric test was used to compare the age distribution between the two subgroups, and χ^2^ tests were used to compare the distribution of other clinicopathological features.

The Kaplan–Meier method with a two-sided log-rank test was used to compare the overall survival (OS) of patients in different subgroups stratified by *TERT* promoter mutant status, *BRAF* V600E mutant status, H3 K27M-mutant status, histological grading, and 2016 WHO grading. Univariate and multivariate Cox regression analyses were performed to determine the association between the clinicopathological features and the OS of patients with histological grade II–IV gliomas. A value of *P* < 0.05 was considered statistically significant.

## Results

### Characteristics of all patients and samples

**Fig. 2:**
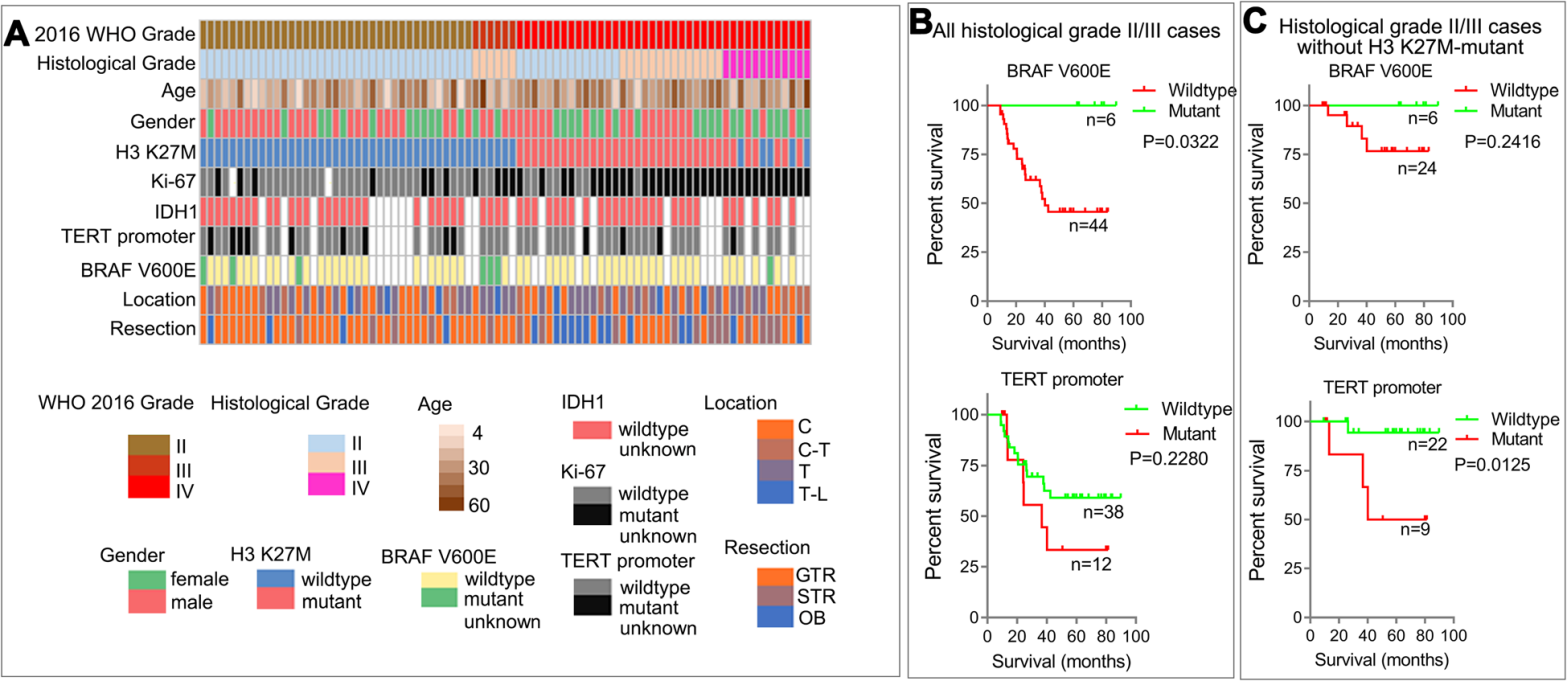
Characteristics and survival rates of spinal cord glioma patients. **a** The landscape of clinicopathological features of spinal cord glioma patients. GTR: gross total resection, ≥ 90%; STR: subtotal resection, ≥ 50% and < 90%; OB: Open biopsy, < 50%. **b**–**c** Kaplan–Meier survival curves of patients stratified by *BRAF* V600E mutation status and *TERT* promoter mutation status in (**b**) all histological grade II/III astrocytomas and (**c**) grade II/III astrocytomas without the H3 K27M mutation

A total of 83 patients who underwent open surgery and were pathologically confirmed to have spinal cord astrocytomas from 2011 to 2018 were enrolled in this study. Their clinical and molecular pathological characteristics are summarized in Table 1. The landscape of the clinical and molecular pathological characteristics of all cases enrolled in this study were also presented, and the 28 histological grade II/III spinal cord gliomas with the H3 K27M mutation were classified as WHO grade IV according to the integrated diagnosis of 2016 WHO classification (Fig. 2a) [24].

The median age at diagnosis of all patients was 31 years (range: 6–63 years), and the median age at diagnosis of patients with H3-wildtype and H3 K27M-mutant astrocytomas was 30 (range: 6–63 years) and 35 years (range: 9–52 years), respectively. The H3 K27M mutation could be observed in histological grade II (40%), III (40%), and IV (20%) astrocytomas, and the distribution ratio of histological grade was significantly different (*P* = 0.0068) between H3 K27M-mutant and H3-wildtype cases. The gross total resection (GTR) number of H3 K27M-mutant astrocytomas (34%) was lower (*P* < 0.0001) than that of H3-wildtype astroctyomas (79%). The percentage of patients with H3-wildtype astrocytomas receiving chemotherapy (21%) was lower (*P* = 0.0484) than that of H3 K27M-mutant glioma patients (37%).

The proportion of Ki-67-positive nuclei was significantly increased (*P* < 0.0001) in H3 K27M-mutant astrocytomas; a total of 28/35 H3 K27M-mutant cases had a high percentage of Ki-67-positive nuclei (≥ 10%), while only 18/48 H3-wildtype cases had a high percentage of Ki-67-positive nuclei. All 58 tested patients lacked mutations in *IDH1* R132H. Furthermore, 7/57 tested patients harbored the *BRAF* V600E mutation, all of whom were classified with H3-wildtype astrocytomas. Additionally, 13/58 tested patients had mutations in the *TERT* promoter, which were identified in both H3-wildtype (9/33) and H3 K27M-mutant (4/25) cases.

Of all 83 cases, 57 cases and 58 cases had *BRAF* V600E- and *TERT* promoter-mutant information, respectively. Initially, we studied the prognostic value of *BRAF* V600E and *TERT* promoter mutations through univariate Cox regression (data not shown) in all cases. Compared to wildtype cases, *TERT* promoter-mutant cases showed a relatively higher HR (1.523, 95% CI: 0.64–3.61), and *BRAF* V600E-mutant cases showed a lower HR (0.189, 95% CI: 0.03–1.40); however, these differences were not significant (*P* = 0.339 and *P* = 0.103, respectively). Considering that the statistical insignificance may be caused by the general poor prognosis of histological grade IV cases with or without these mutations, we next investigated the clinical implications of *BRAF* V600E and *TERT* promoter mutations in all histological grade II/III astroctyomas (Fig. 2b) and histological II/III astrocytomas without the H3 K27M mutation (Fig. 2c). The OS of histological grade II/III glioma patients with the *BRAF* V600E mutation was significantly higher than that of patients without the mutation (*P* = 0.032). In patients diagnosed with histological grade II/III astrocytomas without the H3 K27M mutation, *TERT* promoter mutation was an indicator of poor prognosis (*P* = 0.013).

**Fig. 3:**
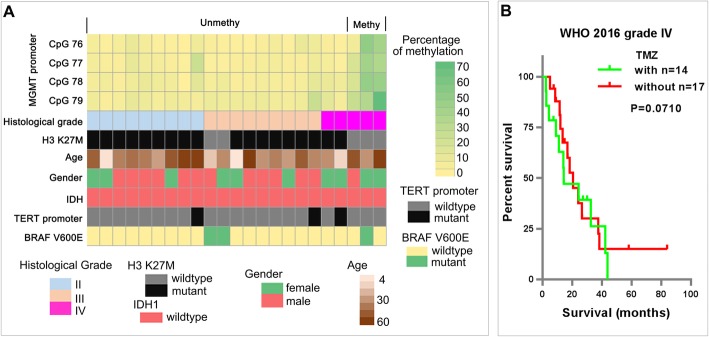
MGMT promoter methylation status and chemotherapy responses of spinal cord astrocytomas. **a** The methylation levels of MGMT promoter CpGs 76–79 and the clinicopathological features of spinal cord gliomas. **b** The impact of TMZ treatment on the survival of patients with WHO 2016 grade IV gliomas

In the 23 gliomas with *MGMT* promoter methylation information, the *MGMT* promoter of all 18 H3 K27M-mutant gliomas were unmethylated, and 3/5 H3-wildtype gliomas had methylated *MGMT* promoters (Fig. 3a). Moreover, there was no significant difference (*P* = 0.710) in the OS of 2016 WHO grade IV glioma patients with (*n* = 14) or without (*n* = 17) TMZ treatment (Fig. 3b).

### The impact of H3 K27M mutation in the prognosis of spinal cord astrocytomas

In all diffuse spinal cord astrocytic tumors, we observed that the median OS of H3 K27M-mutant cases was significantly shorter (*P* < 0.0001) than that of H3-wildtype cases (Table 1). To better understand the prognostic value of the H3 K27M mutation and histological grade, we performed univariate and multivariate Cox regression analyses in all grade II–IV astrocytoma samples (Table 2). Except for gender (*P* = 0.056), all other characteristics, including histological grade (*P* < 0.0001), age (*P* = 0.007), H3 K27M-mutant status (*P* < 0.0001), Ki-67-positive rate (*P* < 0.0001), and resection extent (*P* < 0.0001), affected the prognosis in univariate Cox regression analysis. However, only histological grade [Hazard ratio [HR] = 1.84, 95% confidence interval [CI]: 1.13–3.01, *P* = 0.014] and H3 K27M-mutant status [HR = 2.53, 95% CI: 1.13–5.65, *P* = 0.024] were significantly associated with OS in multivariate Cox regression analysis.

**Table 2:** Univariate and multivariate Cox analysis of clinicopathological features in spinal cord gliomas with WHO 2016 grade II–IV

	Univariate				Multivariate			
	HR	Confidence interval		*P*-value	HR	Confidence interval		*P*-value
		Low 90%	High 95%			Low 90%	High 95%	
**Histological grade**	2.894	1.941	4.315	**< 0.0001**	1.844	1.130	3.009	**0.014**
**Age**	1.031	1.008	1.054	**0.007**	1.024	0.999	1.051	0.060
**Gender**	0.546	0.293	1.016	0.056	–	–	–	–
**H3 K27M**	6.004	3.009	11.980	**< 0.0001**	2.527	1.130	5.652	**0.024**
**Ki-67**	5.660	2.659	12.044	**< 0.0001**	2.253	0.933	5.444	0.071
**Resection**	1.901	1.336	2.707	**< 0.0001**	1.331	0.886	2.001	0.168

*HR* hazard ratio

Significant *P*-values are indicated in bold text

**Fig. 4:**
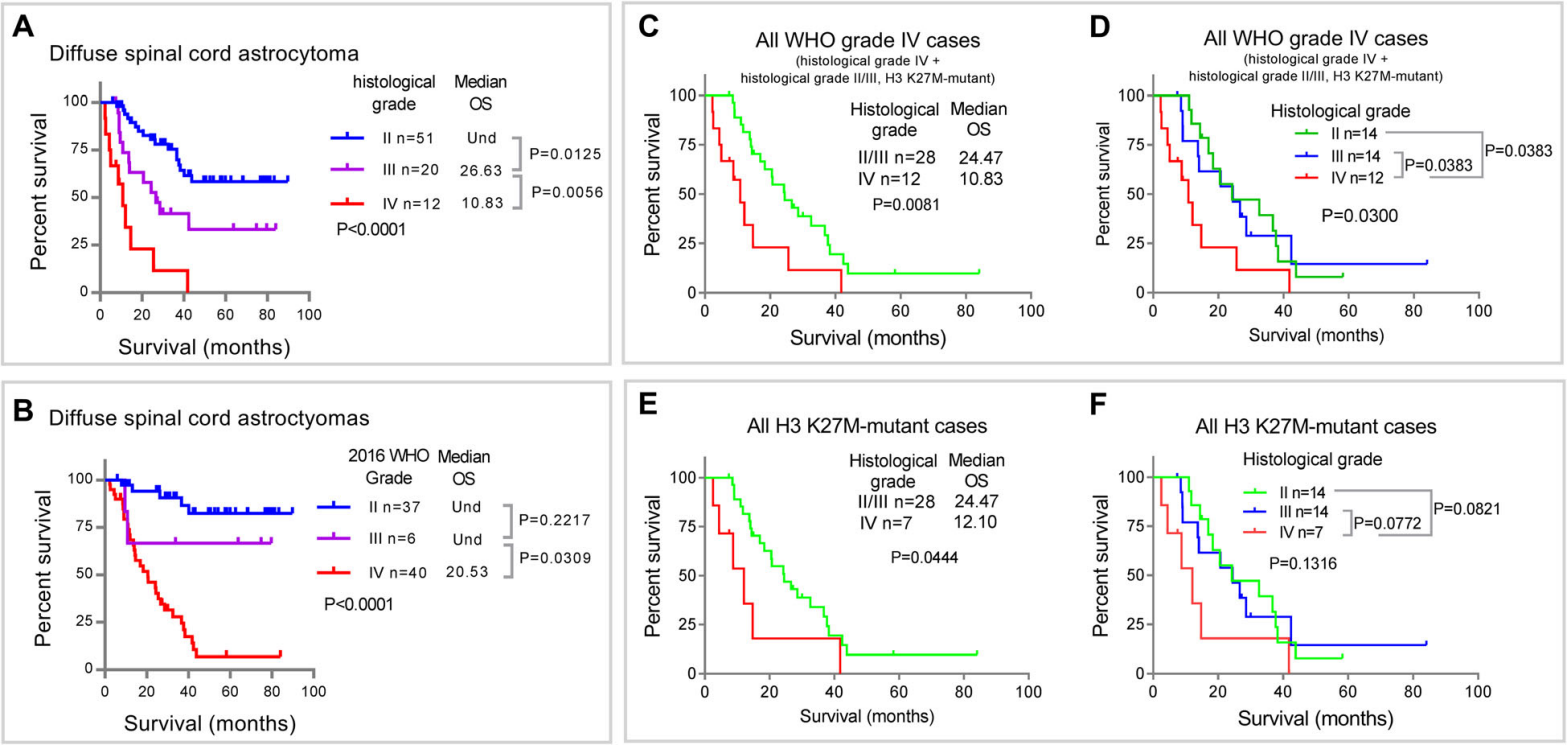
The prognostic value of the H3 K27M mutation in 2016 WHO grade IV astrocytomas (**a**–**b**) Kaplan–Meier survival curves for patients stratified by (**a**) traditional histological grade and (**b**) the integrated diagnosis 2016 WHO grade. **c**–**d** Kaplan–Meier survival curves for patients with all 2016 WHO grade IV astrocytomas stratified by histological grade. **e**–**f** Kaplan–Meier survival curves of patients with H3 K27M-mutant astrocytomas stratified by histological grade

We then compared the OS-stratifying abilities of the traditional histological grading and integrated diagnosis of 2016 WHO classification (Fig. 4a and b), and there were 28 histological grade II/III gliomas with the H3 K27M mutation were classified as WHO grade IV according to the integrated diagnosis of 2016 WHO classification. In general, both the traditional histological grading (*P* < 0.0001) and integrated diagnosis of 2016 WHO classification (*P* < 0.0001) successfully stratified the OS of spinal cord gliomas. However, we noticed that the median OS (20.53 months) of patients with 2016 WHO grade IV astrocytomas, including histological grade IV astrocytomas and H3 K27M-mutant histological grade II/III astrocytomas, was longer than that (10.42 months) of patients with only histological grade IV astrocytomas (Fig. 4a and b).

The above findings suggested that the histological grade maybe still important for accurately diagnosing and predicting the prognosis of H3 K27M-mutant spinal cord astrocytomas. Thus, we directly studied the role of histological grading in all WHO grade IV cases diagnosed by the integrated diagnosis of 2016 WHO classification. We observed that the OS of patients with H3 K27M-mutant histological grade II/III astrocytomas was significantly longer than that of patients with histological grade IV astroctyomas (Fig. 4c and d). In addition, a similar result could be observed in all H3 K27M-mutant astroctyomas (Fig. 4e and f). The OS of histological grade II/III cases was also longer than that of histological grade IV cases (Fig. 4e). A similar tendency was also observed in separate comparisons, with both histological grade II (median OS = 24.23 months) and histological grade III cases (median OS = 24.47 months) surviving better than histological grade IV cases (median OS = 12.10 months), despite the differences were marginal (*P* = 0.082 for grade II vs IV and *P* = 0.077 for grade III vs IV) (Fig. 4f). We speculate that this statistical insignificance is due to the limited number of histological grade IV cases.

### Comparison of the characteristics and prognosis of patients with or without K27M-mutation in each histological grade

The above findings demonstrated that the histological grade cannot be ignored in H3 K27M-mutant astrocytomas, and that the impact of H3 K27M mutation on spinal cord astrocytomas may also differ in astrocytomas with different histological grades. Thus, we also comprehensively compared the clinicopathological features and prognoses of H3 K27M-mutant and H3-wildtype gliomas in each of the grade II, III, and IV samples (Table 3). Compared to patients with H3-wildtype gliomas, H3 K27M-mutant gliomas (*n* = 14) showed a lower ratio of GTR (21% vs 81%, *P* < 0.0001), a higher ratio of Ki-67-positive samples (57% vs 19%, *P* = 0.0108), and a lower survival rate (*P* < 0.0001) only in histological grade II astrocytomas, but not in histological grade III and histological grade IV astrocytomas. There were no significant differences in all characteristics (except for *BRAF* V600E mutation) and prognosis between H3-wildtype and H3 K27M-mutant histological grade III gliomas. The age at diagnosis of H3 K27M-mutant histological grade IV cases was significantly younger (median age 20 vs 51 years, *P* = 0.0101) than that of H3-wildtype histological grade IV cases.

**Table 3:** Comparison of the characteristics and prognoses of histological grade II–IV spinal cord glioma patients with or without the H3 K27M mutation

	Histological Grade II			Histological Grade III			Histological Grade IV		
	H3 wildtype	H3 K27M-mutant	*P*-value	H3 wildtype	H3 K27M-mutant	*P*-value	H3 wildtype	H3 K27M-mutant	*P*-value
Number	37	14		6	14		5	7	
Age (year)	29	36	n.s	30	35	n.s	51	20	**0.0101**
	(6–54)	(10–51)		(13–63)	(9–52)		(30–63)	(13–36)	
Gender			n.s			n.s			n.s
male	25(68%)	7(50%)		4(67%)	9(64%)		2(40%)	2(29%)	
female	12(32%)	7(50%)		2(33%)	5(36%)		3(60%)	5(71%)	
Location			n.s			n.s			n.s
C	16(43%)	5(36%)		1(17%)	3(21%)		2(40%)	2(29%)	
C-T	5(14%)	1(7%)		0(0%)	3(21%)		1(20%)	3(43%)	
T	13(35%)	7(50%)		4(67%)	6(43%)		2(40%)	2(29%)	
T-L	3(8%)	1(7%)		1(17%)	2(14%)		0(0%)	0	
Resection			**< 0.0001**			n.s			n.s
GTR	30(81%)	3(21%)		5(83%)	7(50%)		3(60%)	2(29%)	
STR	3(8%)	3(21%)		1(17%)	3(21%)		2(40%)	4(57%)	
OB	4(11%)	8(57%)		0(0%)	4(29%)		0(0%)	1(14%)	
Radio			n.s			n.s			n.s
yes	25(68%)	9(64%)		4(67%)	10(71%)		1(20%)	4(57%)	
no	8(22%)	3(21%)		1(17%)	3(21%)		3(60%)	0(0%)	
unknown	4(11%)	2(14%)		1(17%)	1(7%)		1(20%)	3(43%)	
Chemo			n.s			n.s			n.s
yes	6(16%)	4(29%)		3(50%)	7(50%)		1(20%)	2(29%)	
no	27(73%)	8(57%)		2(33%)	5(36%)		3(60%)	2(29%)	
unknown	4(11%)	2(14%)		1(17%)	2(14%)		1(20%)	3(43%)	
IDH1			n.s			n.s			n.s
wildtype	26(70%)	11(79%)		4(67%)	10(71%)		3(60%)	4(57%)	
mutant	0(0%)	0(0%)		0(0%)	0(0%)		0(0%)	0(0%)	
unknown	11(30%)	3(21%)		2(33%)	4(29%)		2(40%)	3(43%)	
TERT promoter			n.s			n.s			n.s
wildtype	17(46%)	10(71%)		4(67%)	8(57%)		3(60%)	3(43%)	
mutant	9(24%)	1(7%)		0(0%)	2(14%)		0(0%)	1(14%)	
unknown	11(30%)	3(21%)		2(33%)	4(29%)		2(40%)	3(43%)	
BRAF V600E			n.s			**0.0020**			n.s
wildtype	23(62%)	10(71%)		1(17%)	10(71%)		2(40%)	4(57%)	
mutant	3(8%)	0(0%)		3(50%)	0(0%)		1(20%)	0(0%)	
unknown	11(30%)	4(29%)		2(33%)	4(29%)		2(40%)	3(43%)	
Ki-67			**0.0108**			n.s			n.s
< 10%	28(76%)	6(43%)		2(33%)	1(7%)		0(0%)	0(0%)	
≥10%	7(19%)	8(57%)		4(67%)	13(93%)		5(100%)	7(100%)	
unknown	2(5%)	0(0%)		0(0%)	0(0%)		0(0%)	0(0%)	
Median OS (months)	Und	24.23	**< 0.0001**	Und	24.47	n.s	10.83	12.10	n.s

*C* cervical vertebrae, *C-T* cervicothoracic vertebrae, *T* thoracic vertebrae, *T-L* thoracolumbar vertebrae, *GTR* gross total resection, *STR* subtotal resection, *OB* open biopsy, *Radio* Radiotherapy, *Chemo* Chemotherapy

Significant *P*-values are indicated in bold text

## Discussion

Recent advances in molecular studies, including the identification of the *IDH* mutation, 1p/19q co-deletion, H3 K27M mutation, *EGFR* amplification, *TERT* promoter mutation, *BRAF* V600E mutation, *MGMT* promoter methylation, and others, have revolutionized the diagnosis, classification, and precision chemotherapy of gliomas [5, 8, 9, 14, 19, 24]. However, compared to brain gliomas, previous studies on spinal cord gliomas have usually included less than 30 cases in a single report, and the molecular characteristics of spinal cord glioma (astrocytoma) are still largely unknown [6, 21, 27, 33, 38]. In the present study, we summarized the clinical and basic molecular pathological characteristics of 83 patients with spinal cord astrocytomas, thus revealing the prognostic value of the *BRAF* V600E and *TERT* promoter mutations in grade II and III gliomas. We also performed a direct comparison between H3 K27M-mutant and -wildtype astrocytomas in each histological grade, clarifying that the histological grade is a critical factor for evaluating the impact of the H3 K27M mutation on the prognosis of 2016 WHO grade IV spinal cord astrocytomas.

Due to their sensitive location, the complete surgical removal of spinal cord gliomas is difficult, especially for gliomas with higher histological grades [11, 20, 26, 28, 32]. In our cohort, we noted that gliomas with the H3 K27M mutation had a lower GTR number than H3-wildtype gliomas. However, patients rarely benefit from adjuvant or neoadjuvant chemotherapy and radiation therapy [10, 16]. This may be due to the low rate of *MGMT* promoter methylation in spinal cord gliomas [4, 33, 38]; this was also observed in our study. Moreover, *MGMT* promoter methylation was only identified in H3-wildtype gliomas. Based on this, the development of targeted therapy based on the characteristics of spinal cord gliomas is urgently required.

Although *IDH1* mutations frequently occur in brain astrocytomas, the incidence of *IDH1* mutations in spinal cord astrocytomas has been found to be rather low [2, 35]. No *IDH1* mutations were observed in two previous studies of nine and 17 grade II/III spinal cord astrocytomas [13, 31]. Similarly, *IDH1* mutations were also not observed in another study of 25 cases of 2016 WHO grade IV spinal cord glioma [38]. Here, we confirmed this finding in a larger cohort (*n* = 58) of spinal cord astrocytoma cases with different histological grades. Moreover, we noticed that the median age of H3 K27M-mutant spinal cord astrocytomas was 35 years (range: 9–52 years), and that the median OS of H3 K27M-mutant spinal cord astrocytomas was 20.7 months; this is consistent with previous reports, but significantly different from that of brainstem astrocytomas, which mainly occurs in adolescents and shows a median OS of less than 12 months [10, 18, 21, 28, 33]. Overall, these findings suggest that there are differences in the genetic factors controlling the development of brainstem and spinal cord astrocytomas.

It has been suggested that *TERT* promoter mutations are associated with a poor survival rate in *IDH*-wildtype lower-grade brain gliomas [5, 29, 36]. The similar effect of *TERT* promoter mutation in spinal cord glioma was suggested in three cases [2]. In the present study, we revealed that the rate of *TERT* promoter mutations was 22.4% (13/58); additionally, this mutation was found mainly in H3-wildtype tumors (9/13), and was associated with a poor prognosis for grade II/III spinal cord gliomas. The *BRAF* V600E mutation, *KIAA1549-BRAF* fusion, *FGFR* alterations, and *MYB* or *MYBL1* rearrangements are key prognostic and therapeutic markers for diffuse brain gliomas with wildtype *IDH* and H3 in children or adolescents [3, 14, 15, 30]. Here, we also found that *BRAF* V600E only occurred in H3-wildtype grade II/III spinal cord astrocytomas, and was associated with a good prognosis. These findings suggest that the *BRAF* V600E and *TERT* promoter mutant status should also be included in the molecular testing of spinal cord gliomas, especially for histological grade II or III gliomas.

Although diffuse midline gliomas with the H3 K27M mutation have been suggested to correspond to a grade IV classification, the impact of the H3 K27M mutation on spinal cord astrocytomas may be different from its impact on brainstem glioma [25, 33, 38]. The H3 K27M mutation was reported to be associated with poor survival in two studies with 11 (including six H3 K27M-mutant cases) and 29 (including 14 H3 K27M-mutant cases) spinal cord astrocytoma cases [21, 33]. However, a study of 25 (including 20 H3 K27M-mutant cases) 2016 WHO grade IV spinal cord astrocytomas reported that the H3 K27M-mutant was associated with a better prognosis [38]. Interestingly, both the H3 K27M-mutant and -wildtype astrocytomas in the two former studies included gliomas with different histological grades, while the latter study only included 2016 WHO grade IV spinal cord astrocytomas. These findings suggest that these conflicting results may be related to the histological grade of the tumors.

In present study, we analyzed 14, 14, and 7 cases of H3 K27M-mutant spinal cord glioma with histological grade II, III and IV, respectively. Thus, we could directly compare the H3 K27M-mutant astrocytomas to H3-wildtype astrocytomas of the same histological grade. We confirmed that the impact of the H3 K27M mutation on spinal cord glioma prognosis was influenced by the histological grade. The H3 K27M mutation was associated with a poor survival rate only in histological grade II tumors (*P* < 0.0001), but not in histological grade III (*P* = 0.1297) and IV (*P* = 0.7495) tumors. A similar result has been reported in a study that pooled midline gliomas from different anatomic locations [33]. Moreover, in the 2016 WHO grade IV spinal cord astrocytomas, we noticed that the histological grade could stratify the OS of H3 K27M-mutant gliomas, and that H3 K27M-mutant histological grade II/III gliomas showed significantly higher survival than all histological grade IV gliomas or histological grade IV with the H3 K27M mutation. Altogether, these data that the histological grade is a major contributor to the prognosis of H3 K27M-mutant spinal cord gliomas.

The limitations of this study include its retrospective design, that H3 K27M-mutant status was only determined by immunohistochemistry, and that only the basic molecular features of the tumors were analyzed due to a lack of available samples for adequate analyses. Future studies with large-scale molecular analyses are warranted to confirm the specificity of spinal cord astrocytomas, and to identify novel molecular markers for the clinical management of spinal cord astrocytomas.

## Conclusions

We have identified the molecular features of spinal cord astrocytomas in a relatively large cohort of this extremely rare tumor, revealing that spinal cord astrocytomas were mainly wildtype for *IDH1*, that the *MGMT* promoter methylation rate was low, that the *BRAF* V600E mutation was associated with a good prognosis, and that the *TERT* promoter mutation was associated with a poor prognosis. Importantly, although the H3 K27M mutation is recognized as a robust marker for diffuse midline gliomas, we demonstrated that the histological grade cannot be ignored when assessing the prognosis of H3 K27M-mutant spinal cord astrocytomas. Altogether, our findings provide evidence-based information for the improved management of spinal cord astrocytomas.

## Supplementary information

**Additional file 1 **: **Supplementary Table 1.** Primers used in this study

## Data Availability

The datasets used and/or analysed during the current study available from the corresponding author on reasonable request.
