# Supplementary material for: The molecular characteristics of spinal cord gliomas with or without H3 K27M mutation
Source: Acta Neuropathol Commun. 2020 Mar 30;8:40. doi: 10.1186/s40478-020-00913-w (PMC7106747; doi:10.1186/s40478-020-00913-w)
Supplement: Supplementary file 1 — Additional file 1 : Supplementary Table 1. Primers used in this study [file 40478_2020_913_MOESM1_ESM.docx]

**Supplementary table 1. Primers used in this study**

| Primers | Sequence 5'to3' |
| --- | --- |
| IDH1-amplification F | GCTTGTGAGTGGATGGGTAAAAC |
| IDH1-amplification R | TTGCCAACATGACTTACTTGATC |
| IDH1-Sequencing primer | TGGATGGGTAAAACCT |
| TERT-amplification F | CCGTCCTGCCCCTTCACC |
| TERT-amplification R | GGGCCGCGGAAAGGAAG |
| TERT-Sequencing primer | CGCCCCGTCCCGACC |
| BRAF V600E -amplification F | TGCTTGCTCTGATAGGAAAAT |
| BRAF V600E -amplification R | TCAGGGCCAAAAATTTAATCA |
| BRAF V600E -Sequencing primer | TGATTTTGGTCTAGCTACAG |
| MGMT -amplification F | GTTTYGGATATGTTGGGATAGTT |
| MGMT -amplification R | ACRACCCAAACACTCACCAA |
| MGMT -Sequencing primer | GATATGTTGGGATAGTT |

F: forward primer, R: reverse primer
